# Support for learning under naturalistic conditions

**DOI:** 10.1186/s41235-022-00435-0

**Published:** 2022-09-24

**Authors:** Lucy M. Cronin-Golomb, Patricia J. Bauer

**Affiliations:** Department of Psychology, Emory University, 36 Eagle Row, Atlanta, GA 30322 USA

**Keywords:** Naturalistic learning, Factual recall, Inferential reasoning, Memory integration, Self-derivation, Retrieval practice, Restudy

## Abstract

Educational opportunities occur through naturalistic everyday life experiences (e.g., reading a newspaper, listening to a podcast, or visiting a museum). Research primarily examines learning under controlled conditions, such as in a classroom or laboratory. There is relatively little known about the extent to which adults extract semantic content, beyond factual recall, from naturalistic educational experiences. In the present work, we focused on virtual museum exhibits. The materials were sourced directly from an art history museum. The naturalistic nature of this work stems from the type of content used though an important component of naturalistic learning—motivational processes—was not measured. In each of three experiments, we assessed adult learners’ performance on tests of factual recall, inferential reasoning, and self-derivation through memory integration from naturalistic virtual museum exhibits. In anticipation of the potential challenge associated with learning outcomes under naturalistic conditions, we administered a yoked protocol under which participants had opportunities to engage in retrieval practice (Experiment 2a) or restudy (Experiment 2b) as explicit mechanisms of support for the three tests of learning. In all experiments, participants performed successfully on all three tests of learning; factual recall was the most accessible of the three learning outcomes. There was no difference in performance at the group level across experiments, but there was at the individual level, such that idea units generated during retrieval practice predicted learning outcomes, whereas restudy of those exact idea units did not. The current work provides novel insight into mechanisms underlying adult learning from naturalistic educational opportunities.

## Introduction

Everyday life is infused with a wide variety of naturalistic educational opportunities. For instance, at a doctor’s office you might encounter an educational pamphlet, a podcast might recount daily news, and museums might feature a projection of star patterns. In these situations in particular, effective knowledge base expansion relies on the learner to extract relevant information from varied experiences and resources. Yet prior work assessing learning outcomes focuses primarily on controlled educational experiences, such as those that occur in a classroom or a laboratory (Dart et al., 1999; Esposito & Bauer, 2019). The subset of literature that examines naturalistic educational settings, such as zoos or museums, primarily focuses on probed factual recall (Sweetman et al., 2020) and timing and tracking measures as indicators of learning (Falk & Storksdieck, 2005; Lanir et al., 2017). The developmental literature features implementation of controlled variables such as conversation prompts as support mechanisms for learning under naturalistic conditions (Jant et al., 2014). There also is research on how variables such as visitor motivation, prior knowledge, and exhibition design impact museum visitors’ learning outcomes (Falk & Storksdieck, 2005). However, we have relatively little data on how adults perform on tests of specific learning outcomes beyond factual recall from naturalistic educational experiences. Further, to fully understand naturalistic learning in today’s society, it is critical to appreciate the rising prevalence of online educational opportunities. Online educational experiences are important avenues for learning. For example, virtual museums are arguably more accessible to the everyday learner than in-person exhibits; learners can experience virtual museum exhibits from any location with Internet access.

Research examining online learning focuses on teacher and student attitudes towards online interfaces (Buzzard et al., 2011), ways of supporting self-regulated online learning (Wong et al., 2018), asynchronous online discussions in higher education (Fehrman & Watson, 2021), and use of digital tools to promote on-site learning (Ioannidis et al., 2013). To date, little is known about exactly how and to what extent adults acquire information from online naturalistic educational opportunities.

In the present work, in each of three experiments, we addressed this relative void by testing learning outcomes as resulting from adult learners’ interaction with naturalistic virtual museum exhibits. Importantly, the materials were sourced from an on-campus museum, and thus represent actual educational material an everyday learner may encounter “in the wild.” The specific learning outcomes tested were factual recall, inferential reasoning, and self-derivation through memory integration. With the expectation that learning from such naturalistic environments may prove challenging, in a yoked design, we provided learners opportunities to re-engage with learned material via retrieval practice (Experiment 2a) and restudy (Experiment 2b). As a whole, the present research allows for deeper understanding of learning as an everyday life process.

### Introduction to naturalistic learning

Naturalistic learning, whether online or on-site, occurs outside the structured classroom settings of a school or four walls of a laboratory. Common vehicles are museums, newspaper articles, podcasts, and documentaries, to name a few. Such experiences often are created to educate the general public (Hein, 2009). Especially for learners not engaged in formal educational activities, these experiences are the most common means of acquiring new information. Yet at the same time, they may lack the explicit structure and guidance provided to learners by school-based curriculum or even controlled laboratory materials. That is, though naturalistic settings are often carefully designed to help learners acquire information, the learner decides what to attend to, encode, and ultimately incorporate into the knowledge base. They are not necessarily closely guided by an “other” such as a teacher, researcher, or even the museum curator. Therefore, to understand how learners build their knowledge bases outside as well as inside the classroom or laboratory, it is important to study learning under these more naturalistic conditions. The goal of the current work was not to compare naturalistic learning to controlled learning—controlled learning has been studied at length in classrooms and laboratories. Rather, this research is novel in that it builds on prior work examining learning in controlled settings to incorporate assessments of naturalistic learning outcomes including and beyond factual recall.

It is logical to hypothesize that learning under naturalistic conditions differs from learning in classrooms or laboratories. In addition to differences in the types of materials they may encounter, the way learners approach naturalistic educational environments may differ from how they engage with controlled formal settings. Consider that under formal conditions, learners are given explicit instructions (Segev-Miller, 2004) and asked to engage in direct tasks (Alaagib et al., 2019). For example, in classrooms, instructors provide specific learning goals, and further them with lectures and curated problem sets. Specifically, a teacher might highlight certain words on their PowerPoint slides to indicate their importance to their students. In laboratories, researchers direct participants to memorize sets of target words or text passages. For example, in cued-recall tasks common to memory studies, participants might be asked to retrieve previously seen information associated with a specific target word (Coltheart & Langdon, 1998). In both cases, learners are given explicit direction regarding what they are expected to do, namely, learn material, and the to-be-learned material is identified for them. The net effect is that under formal, controlled conditions, learners engage in overt, directed learning.

In contrast to directed learning situations, in both online and on-site naturalistic educational experiences, learning goals are not made explicit and there is less structure in the materials. That is, visitors to a museum may be given a title for an exhibit or display but they are not directly advised what they are expected to learn from it (Falk & Dierking, 2019). Similarly, they may encounter text placards or recorded voiceovers that provide information about the exhibit, but they may not even read or listen to the information. They also are free to select a subset of available information they found most salient from the whole exhibit (Turgay Zıraman & Imamoğlu, 2020).

Online museum exhibits operate similarly to on-site settings—there may be photographs or videos embedded in a digital space; however, the expectation is that the visitor guides their own educational experience without the presence of overt instructions or explicit learning goals. The net effect is that learners may be less likely to extract the semantic content featured in naturalistic learning experiences. Doing so requires self-direction of attention, which imposes its own demands on learning (Brockett & Hiemstra, 2018; Garrison, 1997; Knowles, 1975). Prior work testing naturalistic learning has probed factual recall (Sweetman et al., 2020), but has not focused on productive memory processes such as inferential reasoning or self-derivation through memory integration. Thus, it is unclear how and to what extent consumers of naturalistic educational experiences acquire and expand upon information from the exhibits they encounter. Further, very little has been done addressing questions of *online* naturalistic experiences. This is an important viewpoint to take, as online resources are typically free and thus accessible to larger subsets of communities. Also, they are rising in prevalence, especially as many community education programs have shifted resources online in response to the COVID-19 pandemic. To address these unknowns, in the present research, in each of three experiments, we assessed what adults learned from virtual museum exhibits. Further, in two of the experiments, we also provided explicit opportunity for re-engagement with previously seen content, with the expectation that successful re-engagement would promote success on the tests of inference and self-derivation through integration in particular.

### Introduction to three learning outcomes

We tested three different learning outcomes from virtual museum exhibits: recall of factual information, generation of valid inferences, and self-derivation of new factual knowledge through memory integration. Of the three, factual recall is the only learning outcome that has been directly examined as a naturalistic learning outcome in prior research (Sweetman et al., 2020). Yet each of these types of learning is called upon in everyday life and each has been studied at length under controlled conditions (e.g., Soto et al., 2019; Taveira-Gomes et al., 2015; Varga & Bauer, 2017). We illustrate the outcomes using examples from the virtual museum exhibits used as stimuli in the present research. First, factual recall questions prompt learners to extract explicit information from an encoding experience. For example, while visiting a Native American footwear museum exhibit, a learner might be exposed to the fact that “*Early European traders introduced seed beads to Native Americans.*” When prompted to answer the question, “*Who introduced seed beads to Native Americans?*” the learner engages in direct factual recall to provide the correct answer, “*Early Europeans*.”

The learning outcomes of inferential reasoning and self-derivation through memory integration require a learner to not only engage with explicitly learned information, as in factual recall, but also to produce information that was never explicitly provided in the educational experience. Inferential reasoning questions require a learner to combine information extracted from the educational experience with information already stored in semantic memory (Seel, 2012). For example, a learner might encode the fact that “*Azurite is blue*” based on a photograph of azurite in an exhibit about color in the classical world. When prompted with the question, “*What color would yellow paint turn to if you mixed it with azurite?*” a learner might infer that the paint would turn green, based on their experience in the exhibit and their prior semantic knowledge that blue and yellow mixed together creates green. This process requires the learner to operate beyond factual recall to mesh newly learned information with already-established knowledge structures.

Self-derivation through memory integration is another such process, though instead of combining exhibit information with prior knowledge, the learner must combine across two pieces of information explicitly available within the exhibit (Varga & Bauer, 2017). For example, in a Native American footwear exhibit, a learner might encode the fact that, “*The strawberry is also called the heart-berry*.” Later within the same exhibit, the learner might read “*The Iroquois believed Sky Woman brought the strawberry to earth with her.*” When asked the question “*According to the Iroquois, who brought the heart-berry to earth?*” the learner can integrate information from the two separate yet related episodes of new learning to self-derive the target answer, “*Sky Woman*.”

### Supportive learning strategies

In the present research, in each of three experiments, we tested the learning outcomes of factual recall, inferential reasoning, and self-derivation through memory integration under the naturalistic condition of virtual museum exhibits. As argued above, we anticipated that the conditions would present challenges to learning of the semantic content embedded in the exhibits (Bitgood, 2009). Specifically, the cognitive exertion required for integrating information (with prior knowledge or across episodes of new learning) may be especially effortful under naturalistic conditions. Accordingly, in two of the three experiments we incorporated support for learning in the form of opportunities to re-engage with previously encountered material. In controlled laboratory studies (Smith & Karpicke, 2014) and in formal classrooms (Dobson et al., 2017; Vojdanoska et al., 2010), a learner’s re-engagement with previously learned material has been found to promote learning outcomes. Re-engagement can occur in myriad ways. Prior work examining re-engagement in naturalistic contexts has focused on on-site social activities, such as engaging in reminiscing conversations (Haden, 2010; Haden et al., 2014) or on-site exhibit-related talk (Pagano et al., 2020). However, conversational prompts are challenging to implement in online environments, especially when visitors are approaching exhibits solo, as is the case in the current work. Additionally, prior work focuses primarily on the benefit of conversations in science museums specifically for child learners, yet the focus of the current work is adult naturalistic learning. Thus, we chose to focus on learning strategies previously implemented and tested in adult populations that could also be used in online contexts. Specifically, we focused on *retrieval practice*, or the active recall of previously learned material*,* and *restudy* of specific, previously seen information. Restudy involves re-reading specific material previously presented during the educational experience. Both retrieval practice and restudy have been found to influence overall outcomes (Krishnan et al., 2017; Miyatsu et al., 2018). We hypothesize that, despite differences in learner approach, re-engagement will support learning outcomes under naturalistic settings. To assess the influence of re-engagement, in Experiment 2a, we provided learners the opportunity to retrieve learned information and in Experiment 2b, we provided the opportunity for restudy, as a means of promoting productive processes under naturalistic conditions. We used a yoked design: participants in 2b restudied exact information generated by a matched participant in 2a. To our knowledge, this is the first study directly comparing retrieval practice and restudy using yoked protocols.

### The current research

In the current research, for all three experiments, we sourced naturalistic educational material (hereafter, stimuli) from virtual museum exhibits created by staff at an art history museum. The exhibits featured three distinct topics: Native American footwear, color in the classical world, and snakes in ancient Egypt. Consistent with the self-directed aspect of naturalistic learning experiences, we did not advise participants that they would be asked test questions until after they visited all exhibits. We used the same exhibits in all three experiments. In all three experiments, we included three different learning outcomes: factual recall, inference, and self-derivation through memory integration.

**Fig. 1:**
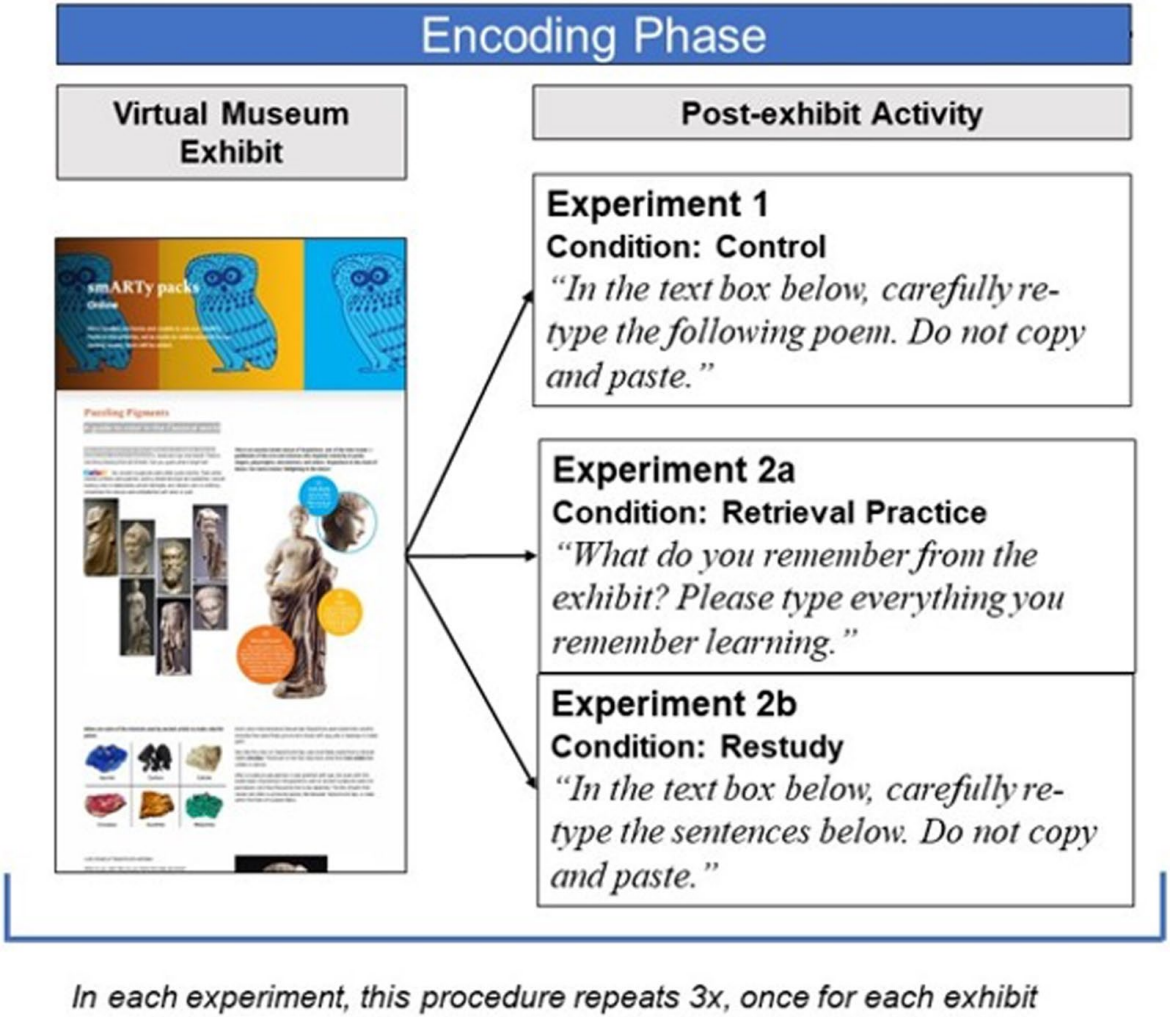
*Schematic illustrating general encoding phase procedure for all three experiments*. *Note* Experiments 2a and 2b are yoked such that the exact content generated by Experiment 2a participants during retrieval practice is presented to participants in Experiment 2b for restudy

As depicted in Fig. 1, each experiment featured a different post-exhibit activity between exposure to the exhibits (encoding phase) and the tests of learning outcomes; the same learning outcomes were tested in each experiment. In Experiment 1, we asked participants to visit each virtual exhibit, engage in an unrelated activity, and then in the test phase, answer questions assessing learning outcomes. The unrelated activity required the same amount of time as the re-engagement activities in Experiment 2 but did not involve re-engagement with the previously experienced material. In Experiment 2a, participants were instructed to engage in retrieval-based re-engagement after they visited each virtual museum exhibit. This manipulation is modeled after retrieval practice used in controlled, formal conditions (Smith & Karpicke, 2014). That is, participants were asked to type a summary of what they learned in the context of the exhibit. In Experiment 2b, participants engaged in restudy-based re-engagement periods. Specifically, each Experiment 2b participant was yoked to an Experiment 2a participant. After visiting each virtual exhibit, Experiment 2b participants retyped the exact responses given by their yoked Experiment 2a counterparts. We designed this manipulation as a way to assess precise differences between retrieval and restudy re-engagement, to assess whether the re-engagement process is as flexible and robust in naturalistic learning environments as it is under controlled conditions. We expected that both retrieval and restudy-based re-engagement periods (Experiments 2a and 2b) would support learning outcomes as compared to lack of opportunity to re-engage (Experiment 1). We also expected that a participant’s success on the retrieval and restudy-based re-engagement periods of 2a and 2b (i.e., how much information they generated or restudied) would influence learning outcomes. As a whole, this research provides novel understanding of learning as it occurs beyond the four walls of a controlled laboratory or classroom.

## Experiment 1

### Method

#### Participants

Participants were 30 adult undergraduate students enrolled in psychology courses at a mid-sized private research university (*M* age = 19.34, 18 females, 12 males). Since there is no research directly examining fact recall, inferential reasoning, and self-derivation through memory integration from naturalistic materials, we estimated a medium effect size. We used G*Power, with 0.8 power, and Cohen’s f effect size of 0.25 and found that for this experiment and all subsequent ones a sample of 30 would be sufficient to test our results. Participants were recruited through SONA research participation software. According to the self-report, the sample was Asian (36.67%), American Indian or Alaskan Native (3.33%), Black or African-American (3.33%), and White or Caucasian (53.33%). 26.67% percent of the sample identified as Hispanic or Latinx. All participants met native English speaker criteria, based on self-report. Two additional participants engaged in protocols but were excluded from the final sample due to self-report of note-taking during learning. Participants were compensated with course credit. Written informed consent was obtained from each participant before the start of their session. For this and subsequent experiments, all study protocols and procedures were approved by the university Institutional Review Board prior to onset of data collection.

#### Stimuli

Stimuli were three thematically distinct, naturalistic virtual museum exhibits. The virtual exhibits were developed by staff at an on-campus art history museum in response to the 2019 coronavirus outbreak. These virtual exhibits were designed to capture information available in the museum’s physical exhibits, to permit community members to engage with rich, salient, educational materials without visiting the museum in person. Importantly, these exhibits are naturalistic in that they were developed prior to current study design, without regard to the goals of the present research. In other words, the exhibits were not developed by researchers with experimental procedures in mind but by museum staff to function as educational tools for the community. As such, they are quintessential “naturalistic” learning materials.

Each virtual exhibit featured both text and photographs and contained thematically distinct information available within each of three different physical museum exhibits. The topics of the exhibits were Native American footwear, color in the classical world, and snakes in ancient Egypt. Virtual exhibits averaged 8 pages (range 7–10), 979 words (range 692–1094), and featured 14 pictures (range 11–19). We did not modify the exhibits from their original design, except to remove interactive elements to which we could not guarantee all participants would have the appropriate software to access (e.g., hyperlinks and games).

#### Procedure

##### Encoding phase

The study description was simply *“In this study, participants will spend time visiting virtual museum exhibits.”* After choosing to participate, participants were sent a link to an online survey built using Qualtrics® software. Upon completion of an online consent procedure, participants were told, *“You will now read through three different exhibits. You have as much time as you need to read through each one.”* Participants then were given the link to the first exhibit and had as much time as needed to read it. Once finished, they closed the exhibit tab and moved to the next page on the survey, where they were told, *“In the text box below, carefully re-type the following poem. Do not copy and paste.”* Participants then re-typed a short poem the content of which was unrelated to any of the exhibits. This post-exhibit activity was timed allowing us to ensure that participants expended effort and did not simply copy and paste the poems. On average, participants spent 2.1 min retyping the poems (range 1.11–4.5 min).

The post-exhibit retyping activity served as a control to the re-engagement procedures of Experiments 2a and 2b. That is, Experiment 1 participants did not re-engage with any material (as they did in Experiments 2a and 2b). Instead, the activity was chosen to match the time elapsed between exposure and test in Experiments 2a and 2b, as well as the motor activity of typing in the subsequent experiments. In order to match the amount of time of the control activity to the re-engagement periods in Experiment 2 protocols, we collected data for Experiments 2a and 2b before Experiment 1. The length of the poem for the control activity was determined based on how long participants in Experiment 2a and 2b spent in re-engagement protocols. Importantly, the design choice of the type of control activity (i.e., a poem) was made before any data were scored or analyzed from Experiments 2a and 2b. After retyping the poem, participants moved to the next page on the survey on which they were given the link to the second virtual museum exhibit. After reading through the second exhibit, participants were asked to re-type another short poem. This process was repeated one more time for the third virtual museum exhibit.

Each virtual museum exhibit was followed by a different poem. Poems were not related to any topics featured in the exhibits and were, on average, 102 words long (range 92–106). The average length of the poems was specifically chosen so participants would expend approximately the same amount of time typing as occurred in the Experiment 2a and 2b samples. The order of poems presented was counterbalanced such that each exhibit was not always followed by the same poem.

Order of virtual museum exhibits was counterbalanced such that exhibits appeared equally often in first, second, and third positions across participants. On average, participants spent 3.32 min visiting each exhibit. A visual depiction of the encoding phase is illustrated in Fig. 1.

##### Open-ended test phase

**Fig. 2:**
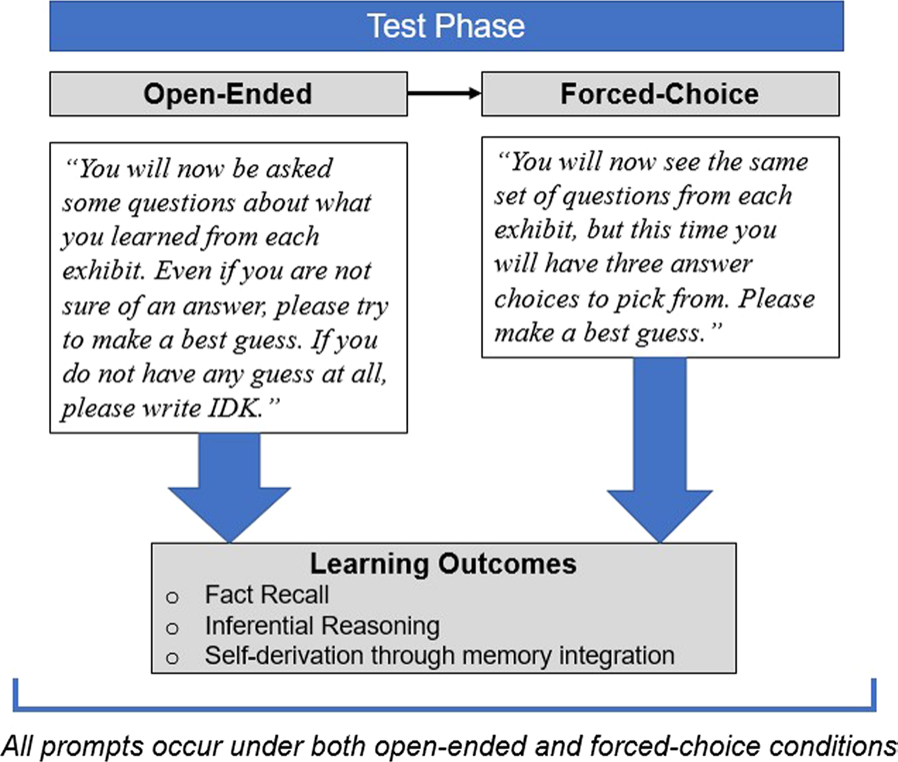
*Schematic of open-ended and forced-choice test phase*

An illustration of the complete test procedure is available in Fig. 2.

After completing a 7-min unrelated buffer activity, participants first engaged with open-ended test questions. They were told *“You will now be asked some questions about what you learned from each exhibit. Even if you are not sure of an answer, please try to make a best guess. If you do not have any guess at all, please write IDK."* Questions were organized in sets such that all questions referencing one exhibit were presented together. Order of question sets was counterbalanced across participants such that each appeared equally often as the first, second, and third test. Within a set, question order was randomized. The order of presentation of the question sets was not linked to the order in which the exhibits were experienced.

Questions were designed to probe three different learning outcomes: factual recall, inferential reasoning, and self-derivation through memory integration. All questions were selected based on pilot data (*N* = 24), to ensure responses to questions were not easily accessible through common prior knowledge. Specifically, pilot test participants were given a battery of questions but were not exposed to the virtual exhibits. Questions that were answered correctly more than 10% of the time under these conditions were not included in the test battery of the study proper. This process ensured that questions in the final test battery assessed learning through the virtual museum exhibits.

Factual recall questions asked participants to retrieve information directly presented in the text of the exhibit. For instance, to correctly answer the question, *“What does the name ‘Mehen’ mean?,”* with the response of “*Coiled one,*” participants needed to extract information directly from the text and photographs of the snakes in ancient Egypt exhibit. Inferential reasoning questions required participants to combine information they learned directly from the exhibit with an element of prior knowledge. For instance, in the Native American footwear exhibit, participants were exposed to the fact that Native Americans often dyed porcupine needles red. When asked, “*What kind of natural materials might Native Americans use to dye porcupine quills?*,” participants needed to first recall the information about the color of quills from the exhibit, then combine it with prior knowledge of red natural materials, and finally infer an answer of some kind of red natural material, such as “*Red berries*.” Self-derivation through memory integration questions required participants to derive information by combining across two pieces of information learned within the exhibit. For example, at one time point in the colors in the classical world exhibit, participants were exposed to the fact that “*A cuirass is made of bronze.”* At another time point within the same exhibit, participants learn, *“Bronze is an alloy made of copper and tin.”* Upon hearing the question *“What two elements make up the metal of cuirass?”* participants needed to integrate across the two separate yet related episodes of within-exhibit learning to derive the correct response, *“Copper and tin.”*

Participants were asked 39 open-ended questions: 14 probed factual knowledge, 14 probed inferential reasoning, and 11 probed self-derivation through integration. There were 3 fewer self-derivation questions than the other two types, due to the nature of the information in the previously designed exhibits. That is, we worked with previously designed stimuli to develop question sets. Self-derivation through memory integration questions require participants to be exposed to two pieces of integrable information, a unique constraint that limited the number of questions that could be appropriately designed under this specific condition. Seventeen questions probed information from the Native American footwear exhibit, 13 were from the color in the classical world exhibit, and 9 were from the snakes in ancient Egypt exhibit. The discrepancies in question number across exhibits were again due to the constraining nature of the previously designed stimulus sets.

##### Forced-choice test phase

After providing answers to all three sets of open-ended questions, participants were told *“You will now see the same set of questions from each exhibit, but this time you will have three answer choices to pick from. Please make a best guess.”* Forced-choice questions provided three answer choices. The two foil choices represented information also available within exhibits, yet inaccurate when matched as a response to the specific questions. As with the open-ended questions, all forced-choice questions were presented as exhibit-based sets. Order of sets were counterbalanced across participants such that each appeared equally often as the first, second, and third set the participant saw. Participants were not necessarily exposed to the question sets in the same order in which they read the exhibits, and they did not necessarily receive the sets in the same order in which they answered them during the open-ended portion. Within set, question order was randomized.

##### Debriefing survey

Upon finishing their session, all participants engaged in a short debriefing survey gauging general feelings towards the experiment, answers to which are available in “Appendix 1,” but which were not formally analyzed.

#### Scoring

For both open-ended and forced-choice tests, participants’ answers were assigned a score of 1 (correct) or 0 (incorrect). Proportion correct across trials were used for analyses. Missing data points were assigned when a participant answered an inferential reasoning or self-derivation question with a factual answer that could technically be considered correct, but which was not the pre-identified target answer to the question. For example, consider the following scenario wherein a participant reads the following two integrable facts: *“The cross-shaped pattern on Arapaho moccasins refers to the four cardinal directions-North, South, East, and West”* and *“The Arapaho people believe the four cardinal directions related to the four “hills of life”: childhood, youth, adulthood and old age.”* When asked the question *“What do the Arapaho people believe the cross-shaped pattern on their moccasin relates to?”* a participant might reply with an answer indicating successful self-derivation through memory integration (e.g., *“The four hills of life*”). But they also might provide an answer indicating factual recall of one fact (e.g., *“The four cardinal directions*”). The factual response is technically an accurate response to the question. Yet it is not the pre-determined target answer indicating self-derivation through memory integration. Thus, it was marked as a missing data point. In Experiment 1, missing data points occurred 1.3% of the time (16 out of 1170 scores were assigned missing data points).

### Results and discussion

**Fig. 3:**
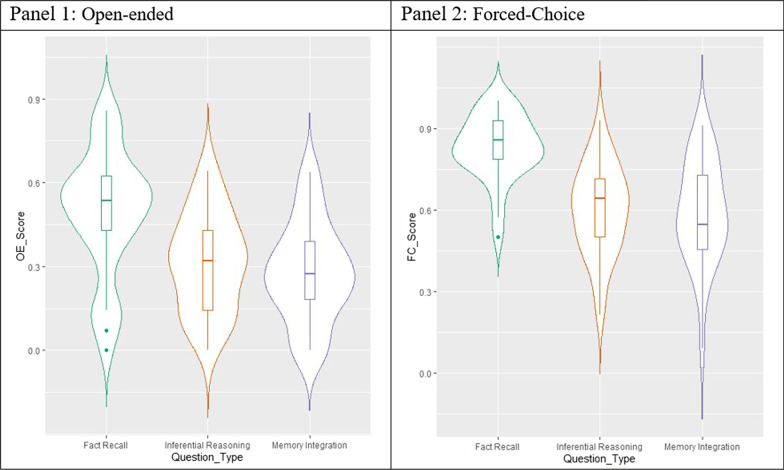
*Open-ended (OE_Score) and forced-choice (FC_Scores) across three question types in Experiment 1*. *Note* Violin plots depict variability and proportion correct for open-ended (OE_Score) and forced-choice (FC_Score) within three question types (fact recall, inferential reasoning, and memory integration)

The main goal of Experiment 1 was to test three learning outcomes from naturalistic virtual museum exhibits, without giving participants the explicit opportunity to re-engage with previously learned material. We first calculated task performance across all three question types (see Table 1). We found striking variability within factual recall (0–85% correct), inferential reasoning (0–64% correct) and self-derivation through memory integration (0–64% correct). Variability and proportion correct within all three question types in Experiment 1 is illustrated in Fig. 3. For this and subsequent experiments, our primary analyses are frequentist in nature. However, to further probe evidence of our null findings, we also include Bayes Factors when appropriate. For all Bayes Factors interpretations, we adhere to the JASP guidelines (van Doorn et al., 2021).

**Table 1:** Learning outcomes in terms of proportion of correct responses from each question type for Experiment 1, Experiment 2a, and Experiment 2b

Experiment	Question type	Open-ended		Forced-choice	
		M (SD)	Range	M (SD)	Range
1	Fact recall	0.49 (0.23)	0.00–0.85	0.83 (0.12)	0.50–1.00
	Inferential reasoning	0.31 (0.18)	0.00–0.64	0.60 (0.17)	0.21–0.99
	Memory integration	0.28 (0.18)	0.00–0.64	0.58 (0.19)	0.09–0.90
2a	Fact recall	0.53 (0.23)	0.00–0.93	0.79 (0.16)	0.29–1.00
	Inferential reasoning	0.35 (0.18)	0.00–0.71	0.63 (0.15)	0.21–0.89
	Memory integration	0.32 (0.17)	0.00–0.60	0.60 (0.17)	0.18–0.90
	Fact recall	0.52 (0.19)	0.07–0.92	0.79 (0.15)	0.43–1.00
2b	Inferential reasoning	0.42 (0.20)	0.00–0.79	0.62 (0.19)	0.21–0.93
	Memory integration	0.33 (0.20)	0.00–0.80	0.63 (0.17)	0.27–0.91

To detect whether there was a significant effect of question type, we conducted separate one-way Type III Sum of Squares Repeated Measures ANOVA using JASP (2021; Version 0.16) for open-ended and forced-choice testing performance. For open-ended testing, there was a significant effect of question type (*F*(2, 87) = 22.64, *p* < 0.001, η_p_^2^ = 0.44). A post hoc Holm–Bonferroni test conducted using JASP software revealed that participants were more successful on open-ended factual recall questions than inferential reasoning (*p* < 0.001) and self-derivation through memory integration (*p* < 0.001) questions, which did not differ from each other (*p* = 0.284). Bayesian post hoc comparisons revealed moderate support for the null finding that inferential reasoning and self-derivation through memory integration did not differ (*BF*_10_ = 0.333).

The same pattern of performance was observed even with the additional support provided by forced-choice options, as depicted in Fig. 3. That is, there was a significant effect of question type (*F*(2, 87) = 32.58, *p* < 0.001, η_p_^2^ = 0.53). A post hoc Holm–Bonferroni test revealed that participants were more successful on forced-choice factual recall questions than inferential reasoning (*p* < 0.001) or self-derivation through memory integration (*p* < 0.001) questions, which did not differ from each other (*p* = 0.683). Bayesian post hoc comparisons revealed moderate evidence for the null finding that inferential reasoning and self-derivation through memory integration outcomes did not differ (*BF*_10_ = 0.208). In forced-choice testing, performance was significantly different from chance (33%) for factual recall (p < 0.001), inferential reasoning (p < 0.001), and self-derivation through memory integration (p < 0.001).

Results suggest that, with naturalistic materials, participants successfully engaged in all three learning processes. Factual information was the most accessible to participants. Inferential reasoning and self-derivation through memory integration might provide a greater cognitive challenge to an individual, as one must incorporate two pieces of information (prior knowledge or within text) to be successful. As such, it is unsurprising that performance was lower on inferential reasoning and self-derivation through memory integration questions, especially in open-ended testing. Yet even with the additional support provided by response options (one of which was correct), the same pattern of performance was observed in forced-choice testing. In Experiment 2, we tested two means to support the productive processes of inferential reasoning and self-derivation through memory integration, namely, retrieval practice and restudy. Of note, these re-engagement activities were designed *in advance* of knowing the results of Experiment 1, based on the logical assumption that learning from naturalistic materials might prove challenging. Indeed, that was borne out by the lower levels of performance in the productive processes of self-derivation through memory integration and inferential reasoning compared to factual recall.

## Re-engagement as support for learning under naturalistic conditions

Re-engagement with previously learned material is an effective strategy of promoting learning outcomes under controlled conditions (Schwieren et al., 2017; Vojdanoska et al., 2010). It is not yet known if re-engagement supports learning from online naturalistic educational materials. It is reasonable to hypothesize that learners, regardless of learning condition, will benefit from an opportunity to re-engage with previously learned material. Learners may encounter unique challenges under naturalistic conditions (Zhu et al., 2020) and as such, may stand to benefit from support mechanisms such as re-engagement. In Experiment 2a and 2b, we focused on retrieval practice and restudy as re-engagement learning strategies to promote learning outcomes, specifically those driven by the productive processes inferential reasoning and self-derivation through memory integration. Fact recall is accessible under online naturalistic conditions (as demonstrated by Experiment 1 results), but processes of inference and self-derivation are less so, even when responses are guided by forced-choice options. The re-engagement protocols of Experiments 2a and 2b elucidate the influence of a re-engagement period on naturalistic learning outcomes. The yoked design of this set of experiments provides novel insight to specific learning mechanisms promoting factual recall, inferential reasoning, and self-derivation through memory integration.

### Yoked design

In Experiment 2a, participants were asked to re-engage with previously learned material through open-ended retrieval practice. This manipulation served three purposes. First, open-ended retrieval practice responses allowed for precise measurement of the amount of information each individual extracted from the naturalistic virtual webpages. That is, participants were asked to record everything they remembered learning from the webpages. These responses could then be broken down into precise units of meaning, which could then be tallied to provide a general measure of amount of learned information (Varga et al., 2022). The second purpose was to provide learners with a mechanism of support for overall learning outcomes. Finally, the third purpose of Experiment 2a was to generate information for the yoked Experiment 2b restudy protocols.

In Experiment 2b, participants were asked to re-engage with previously learned material through restudy of exact information provided by randomly matched Experiment 2a participants. For example, after visiting the Native American footwear exhibit, an Experiment 2a participant wrote, “*I remember that Native Americans used porcupine quills as moccasin decoration*.” After visiting the same exhibit, a randomly yoked Experiment 2b participant restudied (through re-reading and retyping) this exact phrase.

The yoking manipulation served two purposes. The first was to allow for measurements of potential differences between retrieval re-engagement and restudy re-engagement. Participants restudied the information available in the exact retrieval practice responses generated by their yoked Experiment 2a participants. As such, a direct comparison could be made between the influence of retrieval and restudy re-engagement periods on overall learning outcomes. Specifically, we analyzed the correlation of the number of correct units of meaning either retrieved or restudied on overall learning outcomes. For any matched pair of participants, the number of units retrieved or restudied was the same. Thus, any differences found resulted directly from the differences between the processes of restudy and retrieval practice. The second purpose of the manipulation was to provide participants with support mechanism for overall learning outcomes.

## Experiments 2a and 2b

### Method

#### Participants

Experiment 2a participants were 30 adults (*M*age = 21.63, 24 females, 6 males), recruited through Prolific, an online participant recruitment software, due to the beginning of the COVID-19 quarantine. Prolific policy bars collection of individual demographic information beyond gender and age, but allows researchers to set prescreening requirements to permit samples to be customized. All Experiment 2a participants were prescreened such that they were required to be current undergraduate students, who did not have any known literacy difficulties and marked English as their first language. Experiment 2b participants were 30 undergraduate students enrolled in introductory psychology courses at the same university as Experiment 1, recruited through SONA research participation software. Demographic information is only available for 13 of 30 Experiment 2b participants (*M* age = 20, 13 females), due to procedural error. Based on self-report, the available sample was Asian (38.46%), Black or African-American (7.7%), Other (7.7%), and White or Caucasian (30.76%). 15.83% did not report their race. 23.1% of the available sample identified as Hispanic or Latinx. There were no exclusions from Experiment 2a’s sample. Two additional participants engaged in Experiment 2b study protocols but were excluded from the final sample due to experimental error (n = 1), and self-report of note-taking during encoding (n = 1). All participants engaged in an online consent process before beginning their survey and were either compensated $9.50 per hour of their time (Experiment 2a) or with course credit (Experiment 2b). None of the participants had taken part in Experiment 1.

#### Stimuli

The stimuli were identical to those of Experiment 1.

#### Procedure

##### Encoding phase

For both experiments, the study description was simply *“In this study, participants will spend time visiting virtual museum exhibits.”* The procedure was identical to Experiment 1, except instead of retyping a short poem after visiting each museum exhibit, participants re-engaged with previously seen material via *retrieval practice* (Experiment 2a) or *restudy* (Experiment 2b). An illustration of the encoding phase procedure is available in Fig. 1. After visiting the first exhibit, participants moved to the next page of the survey where they were either asked, *“What do you remember from the exhibit? Please type everything you remember learning”*(Experiment 2a) or *“In the text box below, carefully re-type the sentences below. Do not copy and paste”* (Experiment 2b)*.* Participants then had as much time as needed to engage in their respective post-exhibit activity. Experiment 2b participants restudied identical material to that which was generated by participants during the retrieval practice re-engagement portion of Experiment 2a. To elaborate, the researchers randomly yoked each Experiment 2b participant to an Experiment 2a participant.

On average, in Experiment 2a participants spent 2.12 min engaging in retrieval practice (range 0.5–5.9 min), and length of the retrieval practice responses generated was 96 words (range 18–204). In Experiment 2b, each participant spent 2.4 min retyping the sentences from their matched retrieval practice participant (range 1.5–5.0). Participants spent nominally more time retyping than the participants in 2a spent generating information, which gives us confidence that Experiment 2b participants followed instructions and did not simply copy and paste. Participants then moved to the next page on the survey on which they were given the link to the second exhibit. After visiting the second exhibit, participants again engaged in their respective post-exhibit activity. This process was repeated once more for the third exhibit. Order of exhibits was counterbalanced such that exhibits appeared equally often in first, second, and third positions across participants. On average, in Experiment 2a participants spent 4.19 min reading through each exhibit and in Experiment 2b they spent 5.33 min.

##### Test phase

Both open-ended and force-choice protocols were identical to those administered in Experiment 1. A schematic of the test phase is available in Fig. 2.

##### Debriefing survey

Participants engaged in an identical debriefing survey as Experiment 1 (see “Appendix 1”).

#### Scoring

##### Retrieval practice responses (experiment 2a only)

Retrieval practice responses were broken down into their smallest units of meaning. A unit of meaning is a piece of semantic information that can stand on its own (Varga et al., 2022). Units of meaning were tallied within each retrieval practice response. Units of meaning were categorized as “Correct” or “Commentary.” Participants received a “correct units of meaning total score” for each of their three retrieval practice responses, which was the total tally of correct units of meaning, separate from any commentary units. Commentary units included participants’ description of feelings or general side comments. For example, consider the retrieval practice response below:

I remember learning that statues made out of bronze/ change color to green/ because of a process called oxidization. / I thought this was really cool!/ Many of the statues originally had no color/ but were painted using colors from minerals/. However, these did not always stay intact/ and they had to be repainted,/ even though they were protected by a wax coating.

In this response, there are 9 total units of meaning, denoted by slashes. However, the participant only received a “correct units of meaning score” of 8, as the phrase “*I thought this was really cool!*” is categorized as commentary. It does not demonstrate semantic knowledge acquisition. Each “Correct” unit of meaning represents a piece of information that the participant generated that holds its own semantic meaning. Of note, all participants generated information that was accurately derived from the virtual exhibits, thus there was no need for an “Incorrect” coding category. The commentary units generated by 2a participants were also accessible to 2b participants, to ensure the precise amount of retrieved and restudied information was controlled across the two experiments. A second scorer independently coded 25% of the responses. There was 98.46% reliability between the first scorer and the second. The primary scorer’s judgments were used in analyses.

##### Tests

Both open-ended and forced-choice testing was identical to Experiment 1. In Experiment 2a, missing data points occurred 2.7% of the time (32 out of 1170 scores were assigned missing data points). In Experiment 2b, missing data points occurred 1.5% of the time (18 out of 1170 scores were assigned missing data points).

### Results and discussion

**Fig. 4:**
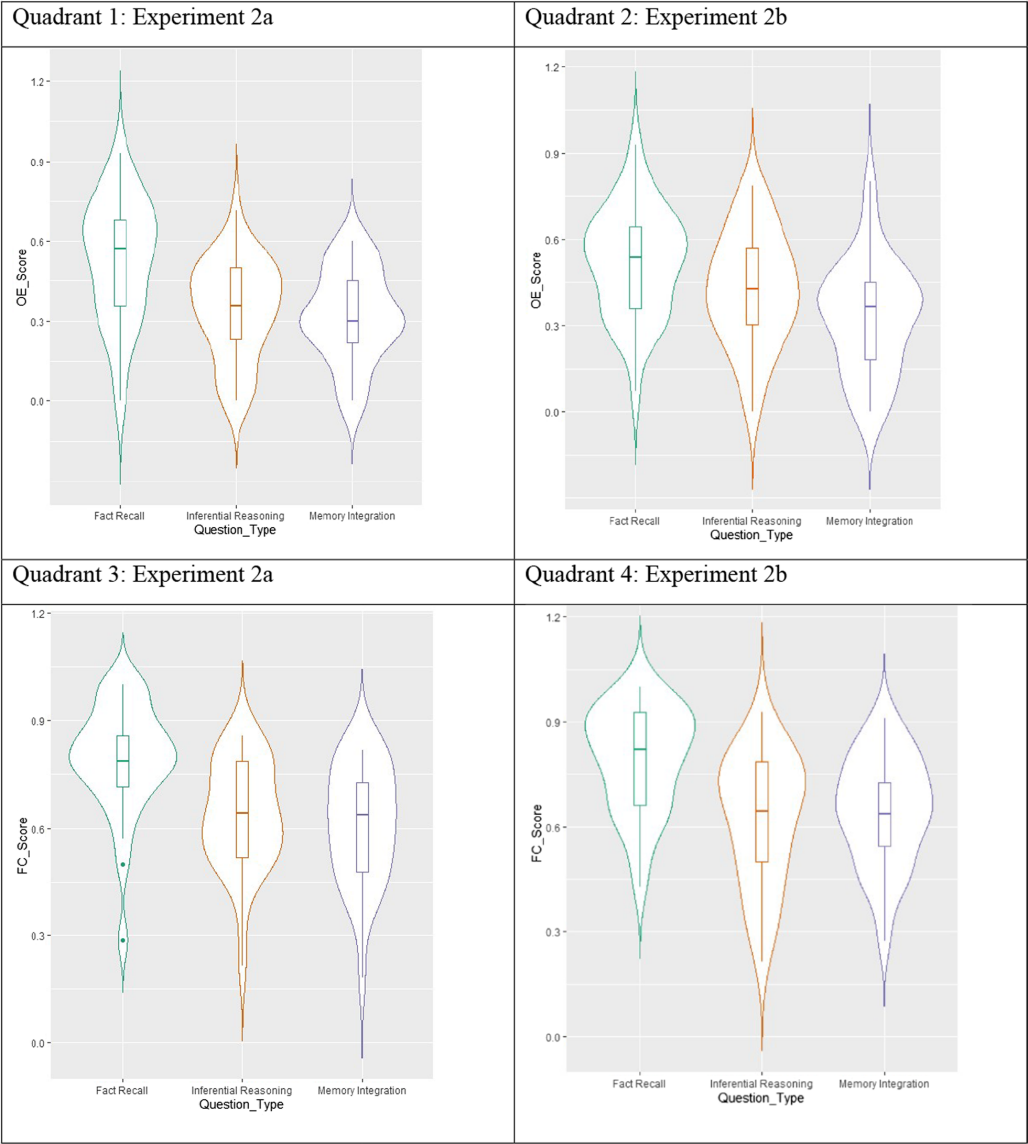
Open-ended and forced-choice learning outcomes across question types in Experiments 2a (Retrieval Practice) and 2b (Restudy). *Note* Violin plots depict variability and proportion correct for open-ended (OE) and forced-choice (FC) performance within three question types (Fact Recall, Inferential Reasoning, and Memory Integration) in Experiment 2a (Quadrants 1 and 3) and 2b (Quadrants 2 and 4)

The purpose of Experiments 2a and 2b was to test the influence of re-engagement as a mechanism of support for learning outcomes from naturalistic virtual museum exhibits, specifically those driven by inferential reasoning and self-derivation through memory integration. To meet this goal, we first analyzed task performance within each experiment. Range, standard-deviation, and proportion correct are reported in Table 1. Variability and proportion correct in open-ended and forced-choice performance in Experiment 2a is illustrated in Fig. 4. We conducted separate one-way Type III Sum of Squares Repeated Measures ANOVAs in JASP for open-ended and forced-choice testing. For both test formats, there were main effects of question type: both open-ended (*F*(2,87) = 24.39, *p* < 0.001, η_p_^2^ = 0.46); forced-choice (*F *(2,87) = 25.28, *p* < 0.001, η_p_^2^ = 0.47). Post hoc Holm–Bonferroni tests revealed that, for both open-ended and forced-choice testing, participants were significantly more successful on factual questions than inference (*ps* < 0.001) or self-derivation through integration questions (*ps* < 0.001), which did not differ from each other (*p*s = 0.414 and 0.213, for open-ended and forced-choice, respectively). Bayesian post hoc comparisons revealed moderate evidence for the null finding that there was no difference in inferential reasoning of self-derivation through memory integration outcomes under open-ended or forced-choice conditions (*BF*_10_s = 0.267, 0.334, for open-ended and forced-choice, respectively). Forced-choice performance was significantly different from chance (33%) for factual recall *(p* < 0.001), inferential reasoning (*p* < 0.001), and self-derivation through memory integration (*p* < 0.001).

We also observed main effects of question type in Experiment 2b, depicted in Fig. 4: open-ended (*F*(2,87) = 20.74, *p* < 0.001, η_p_^2^ = 0.42) and forced-choice (*F*(2, 87) = 25.66, *p* < 0.001, η_p_^2^ = 0.47) conditions. Post hoc Holm–Bonferroni tests revealed that participants in Experiment 2b were more successful on open-ended factual questions than self-derivation through integration questions (*p* < 0.001) and inference questions (*p* = 0.004). Participants were also more successful on open-ended inference questions than self-derivation through integration (*p* = 0.004). As in Experiment 2a, under forced-choice conditions, participants were more successful on factual questions than both inference (*p* < 0.001) and integration (*p* < 0.001), which did not differ from each other (*p* = 0.669). Bayesian post hoc comparisons provided moderate evidence for the null finding that inference and integration did not differ under forced-choice conditions (*BF*_10_ = 0.212). Forced-choice performance was significantly different from chance (33%) for factual recall (*p* < 0.001), inferential reasoning (*p* < 0.001), and self-derivation through memory integration (*p* < 0.001).

In summary, the overall pattern of performance observed in the present experiment was the same as observed in Experiment 1, even though participants in the present experiment were given re-engagement opportunities. These opportunities did not produce the expected differences in proportion correct in the re-engagement groups. We next examined whether individual differences in the extent of re-engagement related to performance at the individual level.

**Fig. 5:**
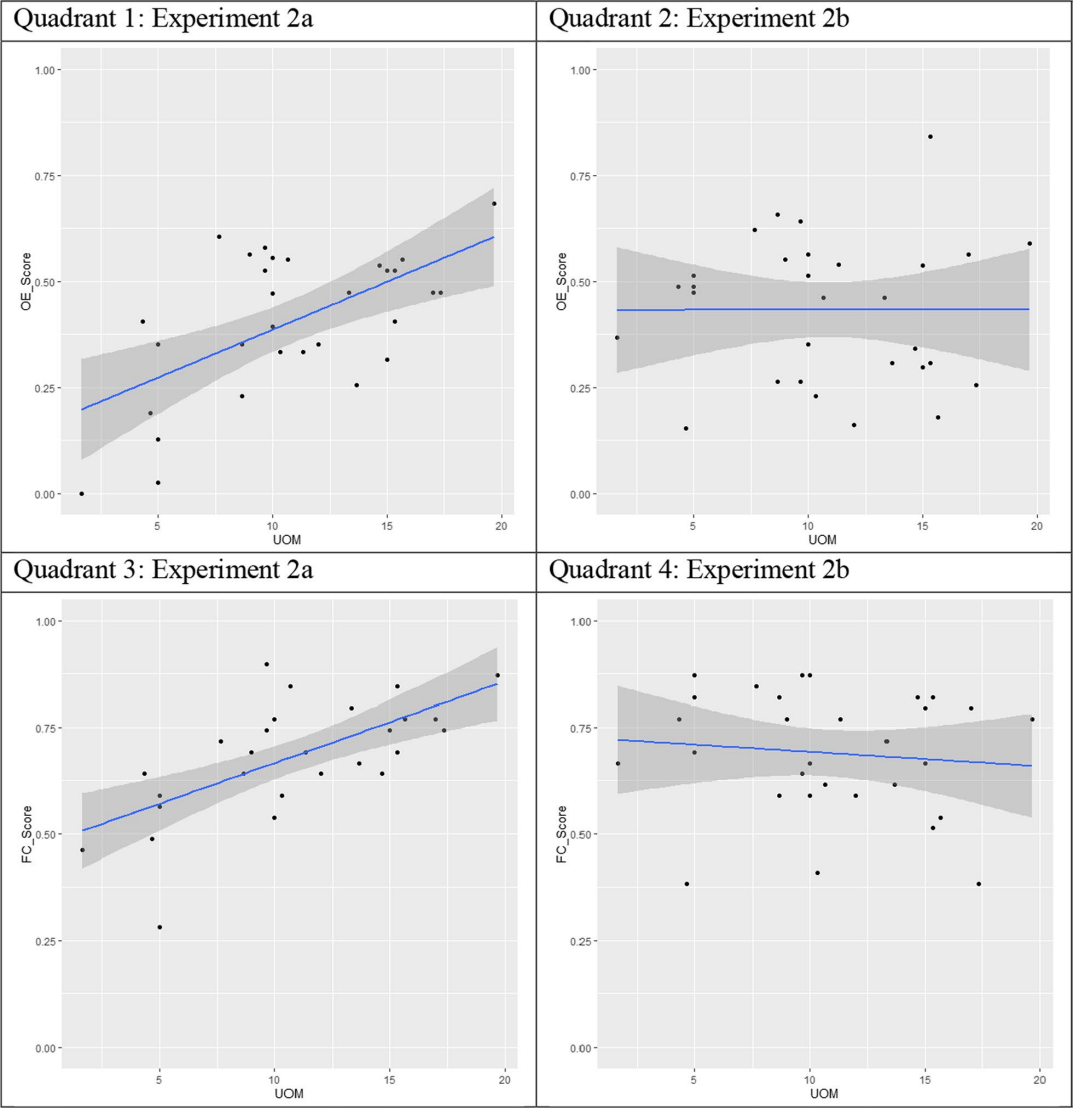
Correct Units of Meaning (UOM) as predictors of open-ended and forced-choice performance in Experiment 2a (Retrieval Practice) and 2b (Restudy)*. Note* Graphs depicting relationship between correct units of meaning (UOM) retrieved (Retrieval Practice Condition; Experiment 2a) and restudied (Restudy Condition; Experiment 2b) and overall learning outcomes under both open-ended (OE; Quadrants 1 and 2) and forced-choice (FC; Quadrants 3 and 4) conditions, when controlling for word count

To examine individual differences between the two types of re-engagement procedures used in Experiments 2a and 2b, we used linear regression models in RStudio (2020; version 1.3.1093), with the lm function in the R Stats package (R Core Team, 2017) for Experiment 2a and then 2b. These models are listed in “Appendix 2.” Performance is illustrated in Fig. 5. Participants in Experiment 2a generated an average of 10.84 correct units of meaning (*SD* = 5.3) and 90.51 words (*SD* = 47.66) during their retrieval-based re-engagement. The number of words produced had a statistically significant effect on both open-ended (β = 0.002, *t* = 2.639, *p* < 0.013) and forced-choice (β = 0.002, *t* = 2.989, *p* < 0.006) performance across learning outcomes. We then added correct units of meaning to the model. Correct units of meaning was a significant predictor for both open-ended (*t*(27) = 3.10, *p* = 0.005, *ΔR2* = 0.21) and forced-choice (*t*(27) = 3.51, *p* = 0.002 0.001, *ΔR2* = 0.24) performance across learning outcomes. Further, once correct units of meaning were added to the model, the effect of word count was no longer significant, suggesting any effect of word count on task performance across learning outcomes was accounted for by correct units of meaning.

We followed the same steps to analyze the influence of correct units of meaning on learning outcomes in Experiment 2b. We first analyzed the influence of word count on task performance across outcomes. There was not a statistically significant effect of word count on open-ended (β = 0.001, *t* = − 0.31, *p* = 0.759) or forced-choice (β = 0.001, *t* = − 0.455, *p* = 0.652) performance. There also was not a significant effect of correct units of meaning for either open-ended (*t* (27) = 0.68, *p* = 0.502, *ΔR2* = 0.02) or forced-choice (*t*(27) = − 0.34, *p* = 0.737, *ΔR2* = 0.004) performance. The effect of word count remained not significant as well. To further probe Experiment 2b’s null findings, we conducted a Bayesian linear regression using JASP. We found that for open-ended outcomes, the best fitting model was the null model (*BF*_M_ = 3.633) compared to the model with both correct units of meaning and word count (*BF*_M_ = 0.296), the model with word count only (*BF*_M_ = 0.650) and the model containing correct units of meaning only (*BF*_M_ = 0.624). This was also the case for forced-choice outcomes: the null model was the best fitting model (*BF*_M_ = 3.549) compared to the model with correct units of meaning and word count (*BF*_M_ = 0.266), word count only (*BF*_M_ = 0.676), and correct units of meaning only (*BF*_M_ = 0.707). These findings are illustrated in Fig. 5.

## Cross-study comparisons

Comparison across the three studies was appropriate, as identical stimuli, test questions, and procedure were used in each sample, except for the re-engagement protocols in Experiments 2a and 2b. Recruitment sources differed across experiments, so we conducted a two-way mixed ANOVA to test whether the main effect of question-type was dependent on experimental cohort. Question-type is within-subject and cohort is between-subject. Cohort is synonymous with experiment sample. For example, all participants in Experiment 1 were grouped in one cohort, all in Experiment 2a were another, and 2b another. We used the aov function in R Studio (see “Appendix 2” for models). Experimental cohort was not significantly related to open-ended (*F*(2,87) = 1.10, *p* = 0.339) or forced-choice (*F*(2,87) = 0.069, *p* = 0.933) task performance. There was a significant effect of question type after controlling for experimental cohort for both open-ended (*F*(2,174) = 65.66, *p* < 0.001) and forced-choice (*F*(2,174) = 82.553, *p* < 0.001) task performance. The interactions between cohort and question type were not significant for either open-ended (*F*(2,174) = 1.24, *p* = 0.297) or forced-choice (*F*(2,174) = 1.47, *p* = 0.215) outcomes. The observed effects of question-type were not influenced by experimental cohort.

We also examined whether performance across virtual museum exhibit differed for each of the three experiments, by conducting a 3 (Experiment) × 3 (Exhibit) mixed ANOVA (Table 2). Experiment is between-subject and exhibit is within-subject. None of the effects were statistically significant (*F*s > 0.01, *p*s > 0.374). Participants’ learning outcomes were not significantly influenced by topic of the museum exhibit.

**Table 2:** Results from mixed model analysis of variance assessing the effects of exhibit (Native American footwear, colors in the classical world, and snakes in ancient Egypt) and experiment on overall learning outcomes

Measures	Open-ended			Forced-choice		
	F	Df	*p*	F	df	*p*
Experiment	0.52	1	0.478	0.047	1	0.830
Exhibit	0.33	1	0.569	0.823	1	0.374
Experiment x Exhibit	0.01	1	0.938	0.073	1	0.790

*p < 0.05; ***p < 0.001

## General discussion

The goal of the current work was to examine adult learning from naturalistic materials experienced in online exhibits. To address this goal, we expanded upon the previous literature on learning outcomes under controlled, directed conditions. To do so, we implemented tests of three learning outcomes from naturalistic virtual museum exhibits, namely tests of factual recall, inferential reasoning, and self-derivation through memory integration across three experiments. In Experiment 1, participants engaged in a short typing activity after exposure to each exhibit. This manipulation served as a control for Experiment 2 protocols. After the typing activity, participants then answered questions testing factual recall, inferential reasoning, and self-derivation through memory integration. Questions probed information available within each exhibit under both open-ended and forced-choice conditions. In Experiment 2, we implemented mechanisms of support for learning, to lessen the potential challenge associated with extraction of information from naturalistic educational experiences. Participants in Experiment 2 were given the opportunity to re-engage with previously seen information after exposure to each exhibit. They re-engaged with material either through open-ended retrieval practice (2a) or restudy (2b). Each participant in 2b restudied exact information generated by their yoked 2a participant during retrieval practice. This yoked design allowed for strong comparison between the specific influences of retrieval-based support compared to restudy-based support on overall learning outcomes.

We first examined performance on the three different tests of learning in each experiment. For all three experiments, performance on factual recall questions was highest under both open-ended and forced-choice conditions. It is viable to speculate that questions of factual recall provide the most direct path for a learner to generate successful response. Correct responses can be extracted directly from educational materials. A learner must encode and retrieve the correct material, but additional steps of memory processes are not required. Generally speaking, there are only two spots where a learner might “fail” when trying to answer a test of factual recall. They might fail to encode the information at the outset, or they might fail to retrieve the correct information when prompted to do so.

In contrast to factual recall, the path to a successful answer on tests of inferential reasoning or self-derivation through memory integration is not as direct. Learners must engage memory processes beyond encoding and retrieval of explicitly available information. For instance, to successfully answer a test of inferential reasoning, a learner must integrate information extracted from an educational experience with a structure of prior knowledge. To do so, they must first recognize the relatedness of the explicitly learned material and their prior knowledge, and then successfully combine across the two to infer an accurate answer. There are many places where a learner can make a misstep in such a process. They might fail to encode and retrieve accurate information, as is the case in tests of factual recall. However, they also might fail to select the correct prior knowledge, fail to recognize the relatedness of items, or even fail to successfully combine across them to make a valid inference. The same challenges might occur during the process of self-derivation through memory integration. To successfully self-derive knowledge, a learner must accurately encode and retrieve two pieces of explicitly available information. They then must combine across the two and derive new factual information. A learner might make a misstep in many places within the self-derivation process, whether it be during encoding, reactivation of relevant information, integration, selection of accurate information, or even during the final step of self-derivation (Bauer & Varga, 2017).

It is unsurprising that factual recall is the most accessible of the three learning outcomes. Indeed, a similar pattern of results has been found under controlled conditions. Performance on self-derivation through integration questions under controlled conditions shows striking variability, whereas performance on questions probing the factual recall of the separate yet related learning episodes necessary for self-derivation is consistently higher (Wilson & Bauer, 2021). Learning under naturalistic conditions requires a discerning eye when separating elements designed to be engaging (e.g., distinctive font, vivid colors), from elements of semantic content. Yet because this challenge occurs during the encoding phase, it is logical to posit that it would have similar influence on performance across all three question types. Thus, it is expected that the most accessible question type under controlled conditions is also the most accessible under naturalistic conditions. Broadly speaking, educational settings, whether controlled or naturalistic, may benefit from increased support for learning through productive memory processes.

An unexpected finding in the present research is that learners performed so similarly across all three experiments. Based on prior work examining mechanisms of support under controlled conditions (Schwieren et al., 2017), we hypothesized that participants who were given the opportunity to re-engage with previously seen material, either through retrieval practice or restudy, would perform higher on all tests of learning (Krishnan et al., 2017). This was not the case. Participants who were not given the chance to re-engage with previously seen material (Experiment 1) performed just as well as those who were (Experiment 2). This effect is salient in our open-ended testing, despite the potential ceiling effects seen in forced-choice performance. One possible reason for this, at least for those participants who engaged in retrieval practice, is that, generally speaking, benefits of retrieval practice have only been found after a delay of at least 48 h (Roediger & Karpicke, 2006), or with particularly challenging test questions (Pyc & Rawson, 2009). In the current work, we administered tests of learning outcomes about 10 min after exposure to educational materials and did not implement a delay period into our protocol. We expected the naturalistic conditions of the learning experience would be sufficiently challenging to generate an effect of retrieval practice, even without implementation of a delay, yet this was not the case. The reasons for the lack of increase in task performance under restudy-based re-engagement conditions are not so clear. Prior work has found restudy to benefit performance on tests of learning administered directly after exposure to educational materials (Roediger & Karpicke, 2006). We did not find evidence of this increase in the current work. To further investigate why task performance did not differ across the three groups, we focused on results from the yoked design of Experiment 2.

The yoked design of Experiment 2 allowed us to specifically test whether the information generated during retrieval practice predicted overall learning outcomes. We found that the amount of correct information generated during retrieval practice predicted learning outcomes, even when controlling for word count. These results suggest that, though as a group, participants who engaged in retrieval practice did not have higher levels of performance compared to the non-re-engagement cohort of Experiment 1, at the individual level, re-engagement experience in the form of retrieval practice facilitated learning. That is, participants who provided more correct information in their retrieval practice responses performed significantly higher on the test questions (see also Varga et al., 2022, for a similar effect under controlled laboratory conditions). This was not the case, however, for the participants who re-engaged in restudy-based protocols. Though these participants restudied the exact information generated by their matched retrieval practice peers, the amount of correct information did not predict performance. A participant who restudied a large amount of information did not necessarily perform better than one who restudied a small amount of information.

We speculate that the process of retrieval itself is the reason behind this pattern of results. That is, it was not the content of the units of meaning, but the actual process of generating the retrieval practice response itself that predicted performance. Retrieval practice is a cognitively demanding task that requires the participant to revisit their own experience and retroactively extract information. For participants who re-read and re-typed the exact units of meaning but did not self-extract them, we did not see any influence on performance, suggesting that it was the actual act of retrieval itself that facilitated learning. In our analyses we included only correct units of meaning as a predictor of learning outcomes, though participants in Experiment 2a also generated commentary units. These commentary units were then also accessible to the matched Experiment 2b participant. We only included correct units of meaning in our analysis for two reasons. First, we were primarily interested in the degree to which retrieving or restudying meaningful semantic content influenced later learning outcomes. Second, of the total 976 codes across the experiment, only 15 were “Commentary,” diluting the potential meaningfulness of any analysis. Though examining the influence of commentary on learning outcomes was outside the scope of the current project, it remains an open question and an important line of future research.

Strikingly, though retrieval practice predicted performance at the level of the individual, we did not find differences at the group level across experiments. The rich, salient nature of the virtual museum exhibits themselves is one possible reason for this surprising finding. To elaborate, the virtual museum exhibits are unique in that they are specifically designed as tools of education for the general public. Within each exhibit there are elements such as bolded words, highlighted phrases, within-text open-ended questions, bright shapes, photographs, cartoons, and maps. Any combination of these elements might prompt the learner to self-generate units of meaning. We only had a measure of units of meaning in Experiment 2a, but that does not mean the participants in the other groups were not inspired by the richness of the materials to spontaneously generate their own meaning, either during encoding, directly after the visit, or even possibly at test. This speculation is supported by the fact that, in all three experiments, there were participants who were successful on nearly all of the test questions (see Figs. 3, 4, 5). A possible direction for future research would be to focus on participant’s direction of attention within virtual museum exhibits (e.g., through eye tracking or mouse-tracking), during re-engagement periods and at test, to assess where exactly each learner is devoting their time and cognitive effort. Eye tracking or mouse tracking methodologies would allow for examination of whether high performers differentially engage with exhibit elements compared to low performers.

Another possible avenue of future research is to examine how and to what extent children extract semantic content from naturalistic educational materials. It is logical to hypothesize that children face greater difficulties learning under such conditions than adults, in part due to lower executive function abilities (Best et al., 2010; Zelazo & Carlson, 2012). Children may struggle to suppress engaging content that is not relevant to semantic content due to potential deficits in inhibitory control (Richland et al., 2006). Additionally, children spend much of their time in classrooms, guided by teachers. Alternatively, it may be that children are more successful in naturalistic settings compared to formal ones. It may be that children have more direct experience acquiring information under informal conditions (e.g., exploratory play) and are more comfortable in this type of free-choice environment than adults. Regardless, naturalistic settings such as museums are places where children can exercise essential skills such as exploration and self-directed learning. It is vital to understand how children learn from such environments, to provide insight into potential ways of maximizing such educational opportunities.

### Limitations

The current research has many strengths and also some limitations. For one, though the materials used in the current work were naturalistic, there were aspects of learning that were not. For instance, we did not have an explicit measure of specific individual motivation for engaging with the virtual museum exhibits. Naturalistic learning is often self-motivated and driven by a variety of situational (e.g., problem solving, educational leisure, strengthening social bonds) and personal (e.g., beliefs in self-efficacy, individual personality characteristics) factors. A limitation of the online, controlled nature of the current work is that participants were compensated with money or course credit; neither factor is naturalistic in nature. Future research should examine not only naturalistic materials but also naturalistic motivations. For example, testing fact recall, inferential reasoning, and self-derivation through memory integration in *in-person* exhibits may provide insight to learning as driven by naturalistic motivational processes.

Additionally, the yoking procedure conducted in Experiment 2a and 2b was somewhat limited in that we did not explicitly match participants based on age, education, or attentional capacities. However, we took care to ensure our two samples represented the same population. That is, samples were customized such that all participants were between the ages of 18 and 22, native English speakers, who were currently enrolled in an undergraduate degree program. We did not expect gender differences and thus did not match based on gender. Matching participants based on attentional capacities is an important direction for future research.

Also, though we did pilot test all test questions to ensure responses required exposure to the virtual museum exhibits, we did not have a direct measure of individual participants’ prior knowledge. How prior knowledge influences learning outcomes is an important line of future research (see Varga et al., 2022). Next, our sample was limited in terms of diversity. It is important to test for replication of these findings in a more diverse sample to ensure the generalizability of the results of this work. Finally, as indicated by the reported Bayes Factors, our data do not provide strong evidence for our null findings. Our assumption of a medium effect size may have overestimated the true effects of differences between the different tests of learning.

### Conclusions

In the present work, we assessed adult learners’ performance on tests of factual recall, inferential reasoning, and self-derivation through memory integration from naturalistic virtual museum exhibits. We implemented explicit learning strategies (i.e., retrieval practice and restudy) to support the three distinct learning outcomes. In all experiments, participants performed successfully on all three tests of learning; factual recall was the most accessible of the three learning outcomes. There was no difference in performance at the group level across experiments. Importantly, there were differences in performance at the individual level, such that correct units of meaning generated during retrieval practice predicted learning outcomes, whereas restudy of those exact units of meaning did not. Additionally, it may be that explicit implementation of learning strategies is not necessary under naturalistic experiences: the rich, salient, engaging nature of the naturalistic stimuli may be enough to encourage learners to engage spontaneously in learning strategies such as retrieval practice.

To our knowledge, this is the first work to date that a) examines learning beyond factual recall using naturalistic materials and b) uses a yoked design to directly control the amount of content explicitly retrieved and restudied across samples, allowing for direct comparison of the influence of the two strategies on overall learning outcomes. As a whole, this work provides novel insight to adult learning as it occurs beyond controlled, directed environments.

## Data Availability

The virtual museum exhibits used in these experiments are available at the following links: Native American footwear, color in the classical world, and snakes in ancient Egypt. None of the experiments were preregistered. The datasets generated during the current study are not publicly available as ongoing projects necessitate the novelty of test questions and forced-choice response options. Data are available from the corresponding author on reasonable request.

## Appendix 1

### Debriefing Survey Responses

Experiment 1 Debriefing Survey Responses (*N* = 30)

**Table:**

Survey question	Mean response score (1 = Definitely Yes, 5 = Definitely No)
Did you learn something new?	1.56
Did you like participating in this study?	2.06
	(1 = Preschool, 5 = Adults)
Which age-group do you think these exhibits were designed for?	3.13
	(1 = Very, 5 = Not at all)
How confident were you in your answer choices during the open-ended portion (no answer choices)?	3.76
How confident were you in your answer choices during the multiple choice portion (three answer choices)?	2.46

Experiment 2a Debriefing Survey Responses (*N* = 30)

**Table:**

Survey question	Mean response score (1 = Definitely Yes, 5 = Definitely No)
Did you learn something new?	1.26
Did you like participating in this study?	1.50
	(1 = Preschool, 5 = Adults)
Which age-group do you think these exhibits were designed for?	3.43
	(1 = Very, 5 = Not at all)
How confident were you in your answer choices during the open-ended portion (no answer choices)?	3.13
How confident were you in your answer choices during the multiple choice portion (three answer choices)?	2.20

Experiment 2b Debriefing Survey Responses (*N* = 30)

**Table:**

Survey question	Mean response score (1 = Definitely Yes, 5 = Definitely No)
Did you learn something new?	1.46
Did you like participating in this study?	2.3
	(1 = Preschool, 5 = Adults)
Which age-group do you think these exhibits were designed for?	3.20
	(1 = Very, 5 = Not at all)
How confident were you in your answer choices during the open-ended portion (no answer choices)?	3.60
How confident were you in your answer choices during the multiple choice portion (three answer choices)?	2.46

## Appendix 2

### Models used for units of meaning analysis in Experiment 2a and 2b

**Model 1**

- OE_Score ~ Correct_UOM + Word_Count

**Model 2**

- FC_Score ~ Correct_UOM + Word_Count

### Models used for two-way ANOVA in cross-study comparisons

**Model 1**

- OE_Score ~ Question_Type*Cohort + Error(Participant/Question_Type)

**Model 2**

- FC_Score ~ Question_Type*Cohort + Error(Participant/Question_Type)
